# A dose escalation and pharmacokinetic study of biweekly pegylated liposomal doxorubicin, paclitaxel and gemcitabine in patients with advanced solid tumours

**DOI:** 10.1038/sj.bjc.6603832

**Published:** 2007-06-05

**Authors:** V Bozionelou, L Vamvakas, P Pappas, S Agelaki, N Androulakis, A Kalykaki, M Nikolaidou, N Kentepozidis, S Giassas, M Marselos, V Georgoulias, D Mavroudis

**Affiliations:** 1 Department of Medical Oncology, University General Hospital of Heraklion, Crete, Greece; 2 Department of Pharmacology, School of Medicine, University of Ioannina, Ioannina, Greece

**Keywords:** doxorubicin, gemcitabine, paclitaxel, pharmacokinetics

## Abstract

To determine the maximum tolerated doses (MTDs) and dose-limiting toxicities (DLTs) of pegylated liposomal doxorubicin (PLD), paclitaxel (PCX) and gemcitabine (GEM) combination administered biweekly in patients with advanced solid tumours. Twenty-two patients with advanced-stage solid tumours were treated with escalated doses of PLD on day 1 and PCX plus GEM on day 2 (starting doses: 10, 100 and 800 mg m^−2^, respectively) every 2 weeks. DLTs and pharmacokinetic (PK) parameters of all drugs were determined during the first cycle of treatment. All but six (73%) patients had previously received at least one chemotherapy regimen. The DLT dose level was reached at PLD 12 mg m^−2^, PCX 110 mg m^−2^ and GEM 1000 mg m^−2^ with neutropaenia being the dose-limiting event. Of the 86 chemotherapy cycles delivered, grade 3 and 4 neutropaenia occurred in 20% with no cases of febrile neutropaenia. Non-haematological toxicities were mild. The recommended MTDs are PLD 12 mg m^−2^, PCX 100 mg m^−2^ and GEM 1000 mg m^−2^ administered every 2 weeks. The PK data revealed no obvious drug interactions. Biweekly administration of PLD, PCX and GEM is a well-tolerated chemotherapy regimen, which merits further evaluation in various types of solid tumours.

Chemotherapy regimens combining paclitaxel (PCX) with doxorubicin have shown high antitumour activity in breast, ovarian, AIDS-related Kaposi's sarcoma and other types of tumours. However, their combination has been associated with severe haematological and cardiac toxicity in patients with metastatic breast cancer (MBC) (Gianni *et al*, 1995; Gehl *et al*, 1996; Sparano *et al*, 1999). Indeed, up to 50% of patients receiving the PCX/doxorubicin combination developed a reduction in the left ventricular ejection fraction (LVEF) below the normal level and 20% of them developed congestive heart failure (Gianni *et al*, 1995; Gehl *et al*, 1996). Several studies have suggested a sequence-dependent tolerability because of altered pharmacokinetics (PKs) when PCX precedes doxorubicin administration (Holmes *et al*, 1996; Gianni *et al*, 1997).

In an effort to reduce toxicity while maintaining the same level of activity, doxorubicin has been entrapped in liposomes. Pegylated technology represents a favourable drug carrier system, since stealth liposomal drugs have a reduced clearance with prolonged circulation half-life. The size and structure of stealth liposomes prevent drug extravasation, resulting in selective drug accumulation in tissues with increased vascular permeability, such as tumour tissues, whereas its concentration in the cardiac muscle remains low (Working *et al*, 1994). Caelyx or Doxil (pegylated liposomal doxorubicin (PLD)) is a long-circulating pegylated liposome-containing doxorubicin, which has been developed to target drug delivery to cancer cells, while reducing the toxicities associated with the free doxorubicin. Clinical trials have shown that PLD has significant activity in various types of tumours, with dose-dependent, cumulative and reversible palmar-plantar erythrodysesthesia (PPE) and mycositis being the main dose-limiting toxicities (DLTs) (Uziely *et al*, 1995; Muggia *et al*, 1997), while myelosuppression, nausea, alopecia and cardiotoxicity are less common and severe compared to free doxorubicin (Berry *et al*, 1998; Safra *et al*, 2000).

Gemcitabine (GEM), a novel S-phase-specific cytidine nucleoside analogue of deoxycytidine, has been shown to have a broad antitumour activity against breast, lung, ovarian, bladder and pancreatic cancer (Merriman *et al*, 1996). Its toxicity profile is acceptable, with the main toxicity being mild myelosuppression of short duration. In general, GEM's favourable single-agent activity and novel mechanisms of action, in addition to its largely non-overlapping toxicities, have facilitated its combination with a variety of chemotherapy agents, including the taxanes. Several phase I and II trials have reported impressive activity for the GEM/taxane doublet with a suggestion of clinical synergism between these two classes of agents (Colomer, 2004). A recent phase III randomised trial showed a clear advantage for GEM plus PCX over PCX alone, as first-line treatment of MBC, in time to disease progression, objective response rate and overall survival (Albain *et al*, 2004). In addition, the GEM/anthracycline (epirubicin, doxorubicin, PLD) combination seems to be well tolerated with promising activity in solid tumours (Rivera *et al*, 2003; Fabi *et al*, 2006).

Chemotherapy regimens combining PCX with PLD, PCX with GEM and GEM with PLD have shown antitumour activity and favourable non-overlapping toxicity in various tumours (Androulakis *et al*, 2002; Mavroudis *et al*, 2002). In addition, the triplet combination of PCX, epirubicin and GEM was active against MBC, but it was associated with a high (about 60%) incidence of grade 3 and 4 neutropaenia and, to a lesser extent, cardiotoxicity (Sanchez-Rovira *et al*, 2000; Conte *et al*, 2001; Cappuzzo *et al*, 2004; Zielinski *et al*, 2005). In order to improve the toxicity profile of the triple combination of PCX plus PLD plus GEM by reducing the dose-dependent toxicities and allowing sufficient time to recover between treatments, a biweekly regimen was developed and evaluated in a phase I study.

## PATIENTS AND METHODS

### Patient selection

Patients with histologically or cytologically confirmed advanced-stage solid tumours, for which there is no effective therapy or who had relapsed after receiving the ‘standard’ first-line treatment, were enrolled onto the study. Prior surgery, radiotherapy (to <20% of bone marrow containing bones) or chemotherapy (maximum two prior regimens) were allowed, but a treatment-free interval of at least 4 weeks was required before entering the study. Other inclusion criteria were: age >18 years, a World Health Organization (WHO) performance status of 0–2, a life expectancy of at least 3 months, an adequate bone marrow (absolute neutrophil count >1500 dl^−1^, Hb >10 g dl^−1^, platelets >100 000 dl^−1^), renal (serum creatinine <1.5 mg dl^−1^), liver (total bilirubin <1.5 mg dl^−1^ and SGOT/SGPT <2 times the upper normal values) and cardiac (normal baseline LVEF by multiple gated acquisition (MUGA) scan or echocardiogram) function and pre-existing peripheral neuropathy ⩽grade (WHO) 1. Additional inclusion criteria were: absence of an active infection or severe malnutrition (loss >20% of the body weight during the last 3 months) and absence of any psychological or social condition potentially hampering compliance with the study protocol. Patients with brain metastases were allowed to participate if they had been irradiated with clinical and/or radiographical improvement, while patients with prior history of congestive heart failure or active and uncontrolled coronary disease were not eligible. The presence of measurable disease was not required. All patients gave written informed consent to participate in the study, which has been approved by the Ethics and Scientific Committees of our Institution.

### Treatment plan

Escalated doses of PLD (Caelyx; Schering Plough Pharmaceuticals, Kenilworth, NJ, USA) (starting dose: 10 mg m^−2^ with increments of 2 mg m^−2^) were administered as a 30-min intravenous (i.v.) infusion on day 1, and PCX (Taxol; Bristol-Myers Squibb Co., Princeton, NJ, USA) (starting dose: 100 mg m^−2^ with increments of 10 mg m^−2^) as a 3-h i.v. infusion followed by GEM (Gemzar; Eli Lilly, Indianapolis, IN, USA) (starting dose: 800 mg m^−2^ with increments of 200 mg m^−2^) as 30-min i.v. infusion on day 2. The regimen was repeated every 2 weeks without growth factor support in cycles of 4 weeks. Premedication for PCX consisted of dexamethasone 20 mg orally 14 and 7 h before treatment, ranitidine 300 mg i.v. and diphenhydramine 50 mg i.v. 30 min before treatment. The prophylactic anti-emetic regimen included ondansetron 16 mg and dexamethasone 8 mg given i.v. 30 min before chemotherapy. The treatment was administrated on scheduled days if the absolute neutrophil count was >1500 dl^−1^, platelets >100 000 dl^−1^, and all the other toxicities had resolved to grade ⩽1. Otherwise, treatment was postponed until the resolution of all toxicities and, then, was restarted with dose reduction at the previous dose level. Doses were also reduced at the previous dose level in case of febrile neutropaenia or platelet transfusion. Patients requiring more than 2 weeks treatment delay for any reason or experiencing a decrease of the LVEF >15% below the baseline values with or without clinical signs of congestive heart failure were withdrawn from the study. Patients who developed DLTs at any cycle of the treatment, received the same treatment but dosed at the previous dose level. Patients continued treatment until prohibitive toxicity, disease progression, achievement of maximal response or consent withdrawn.

### Dose escalation

The following dose levels (mg m^−2^) for the PLD/PCX/GEM combination have been evaluated: 10/100/800, 12/100/800, 12/100/1000 and 12/110/1000. No intrapatient dose escalation was allowed. At least three patients were enrolled at each dose level. If DLT was observed in one of the three patients, three additional patients were treated with the same doses. All patients were assessed for dose-limiting events during the first chemotherapy cycle. The DLT was defined as the occurrence of any of the following events: grade 4 neutropaenia or thrombocytopaenia, febrile neutropaenia, any grade ⩾3 non-haematological toxicity except for nausea/vomiting and any treatment delay >2 days due to unresolved haematological or non-haematological toxicity. If at least 50% of the patients at a certain dose level experienced DLT, the study was completed and the maximum tolerated dose (MTD) level, which is recommended for further phase II studies, was the previous level before the DLT dose level (Socinski *et al*, 2001).

### Patients' evaluation

Baseline evaluations included: patient history, physical examination, complete blood count with differential and platelet count, serum chemistry, chest X-rays, electrocardiogram, echocardiography or MUGA scan with LVEF measurement, thorax and abdomen computed tomography scans and whole body bone scintigraphy. Complete blood counts were performed weekly for all patients or in case of grade 3 and 4 haematological toxicity daily until recovery. Serum chemistry as well as a detailed toxicity questionnaire and a physical examination were performed before each treatment administration. The LVEF was measured at baseline and every 3 chemotherapy cycles in percentage (%) (normal values >45%). Disease status was assessed every 3 cycles or earlier in case of clinical evidence of disease progression. Toxicities were graded according to the National Cancer Institute (NCI) Common Toxicity Criteria version 2.0, and evaluation of response was performed according to the WHO criteria (Miller *et al*, 1981). All patients receiving at least one cycle of treatment were evaluable for toxicity and patients with bidimensionally measurable disease receiving at least three chemotherapy cycles were evaluable for response. After treatment, patients were followed monthly until disease progression by physical examination, blood tests, serum chemistry and any other test that the responsible physician considered necessary.

### Pharmacokinetic study

Samples for measurement of plasma levels of doxorubicin, PCX and GEM were obtained during cycle 1. Blood samples (5 ml each) were taken from an antecubital vein contralateral to the site of injection. Sampling for PLD was set before drug administration and at 1, 6, 24, 72 and 168 h after the beginning of infusion. PCX administration started on the second day of the treatment followed by GEM infusion. Samples for PCX were collected before and at 1, 3, 4, 6, 10 and 24 h after the beginning of drug infusion. GEM sampling was performed in tubes containing tetrahydrouridine before drug administration, at 30, 45 min and 1, 1.5, 4.5 h after the beginning of GEM infusion.

Doxorubicin plasma samples were measured using a reverse-phase high-performance liquid chromatographic (HPLC) method according to Gabizon *et al* (1994). Briefly, 400 *μ*l isopropanol, 400 *μ*l chloroform, 0.5 g ammonium sulphate and internal standard (daunorubucin) were added to 400 *μ*l of each plasma sample. The mixture was centrifuged at 10 000 r.p.m. for 15 min and the supernatant evaporated to dryness under a stream of nitrogen at 30°C. Concentrated samples were reconstituted in 200 *μ*l isopropanol and 50 *μ*l was injected into the HPLC within 24 h. The mobile phase consisted of acetonitrile : water (4 : 6 v v^−1^) adjusted to pH 2.60 with perchloric acid. Analysis was performed on a reverse-phase Lichrospher RP-8 column (150 × 4.6 mm, 5 *μ*m MZ-Analysetechnik GmbH, Mainz, Germany). Doxorubicin was monitored at 470 nm excitation and 590 nm emission wavelengths with fluorescence detector. Standard calibration curve was linear in the range 0.10–12.50 *μ*g ml^−1^ (*r*^2^⩾0.998) with a lower limit of quantitation (LOQ) of 0.05 *μ*g ml^−1^.

For PCX measurements, 100 *μ*l of internal standard (docetaxel) and 5 ml of acetonitrile : *n*-butylchloride (1 : 4 v v^−1^) were added to 1 ml of human plasma based on a method described by Sparreboom *et al* (1998). The organic layer was collected and evaporated to dryness under a stream of nitrogen at 60°C. The residue was reconstituted in 125 *μ*l of methanol : water (1 : 1 v v^−1^) and ultrasonicated for 1 min. A 100 *μ*l portion of the solution was injected to HPLC system. The chromatographic analysis was achieved in an Inertsil ODS-80A column (150 × 4.6 mm, 5 *μ*m; GL Science Inc., Tokyo, Japan) at 60°C. Mobile phase was a solution of water : methanol : tetrahydrofuran : ammonium hydroxide (37.5 : 60 : 2.5 : 0.1 v v^−1^) adjusted to pH 6.0 with formic acid. Flow rate was set at 1 ml min^−1^ and detection of PCX was achieved at 230 nm with an ultraviolet detector. Calibration curve was prepared in blank human plasma with standard concentrations of PCX over the range of 0.01–2.0 *μ*g ml^−1^ (*r*^2^⩾0.9993). The LOQ was determined at 0.01 *μ*g ml^−1^.

Measurements of GEM plasma samples were performed with a reverse-phase HPLC method, as described previously (Mavroudis *et al*, 2003). GEM was assayed in a reversed-phase column *μ*-Bondapack C18 (300 × 3.9 mm, 10 *μ*m Waters, Milford, MA, USA) and monitored at 267 nm with ultraviolet detector. The linear range of the assay was established at 0.1–10 *μ*g ml^−1^ (*r*^2^⩾0.9998) and the LOQ was set at 0.078 *μ*g ml^−1^ of plasma.

Doxorubicin, PCX and GEM were assayed on an LC-10A/10Avp Shimadzu chromatographic system (Shimadzu Deutchland GmbH, Duisburg, Germany) equipped with an RF-10Axl fluorescence detector and an SPD-M10Avp ultraviolet detector. PK parameters for doxorubicin, PCX and GEM were estimated by the non-compartmental method using WinNonlin (Standard edition version 2.1) program (Pharsight Co., Palo Alto, CA, USA).

## RESULTS

### Patients' demographics

From October 2002 to January 2004, 22 patients with advanced-stage solid tumours were enrolled onto the study. All patients were evaluable for toxicity. Median age was 64 years, the performance status (WHO) was 0–1 in 91% of patients, and 16 (73%) of them had received, at least, one prior chemotherapy regimen. Patients' characteristics are shown in Table 1.

### Dose-limiting toxicities

The administration of two biweekly consecutive treatments (4 weeks) was considered as one treatment cycle. Table 2 shows the dose escalation levels, the number of patients enrolled at each dose level and the observed DLTs during the first cycle of treatment. The main toxicity observed during the first chemotherapy cycle was neutropaenia; indeed, two patients developed grade 4 neutropaenia and five patients grade 3 (*n*=2 patients) or grade 2 (*n*=2 patients) neutropaenia and grade 2 (*n*=1 patient) anaemia leading to treatment delay for more than 2 days. At the dose level IV where an additional patient was enrolled because of an initial doubt about the characterisation of toxicity, three out of four patients developed DLTs (one patient grade 4 neutropaenia and two patients treatment delays because of grade 2 and 3 neutropaenia), and therefore, this was considered as the DLT level. The MTDs which are the doses recommended for future phase II studies were PLD 12 mg m^−2^ on day 1 and PCX 100 mg m^−2^ followed by GEM 1000 mg m^−2^ on day 2 every 2 weeks without growth factor support. Three additional patients were treated at the MTD dose level without the occurrence of any additional dose-limiting event (Table 2).

### Haematological and non-haematological toxicities

Eighty-six chemotherapy cycles were administered with a median of three cycles/patient (range, 1–9). Twenty-one (24%) cycles were delayed because of haematological toxicity (*n*=9 cycles; 10.5%) and late admission for reasons unrelated to the disease or treatment (delays for imaging evaluations, *n*=12; 14%). Table 3 shows the number of chemotherapy cycles complicated with grade 2–4 toxicities and Table 4 shows the worst grade 2–4 haematological and non-haematological toxicities per patient by dose-level, during all cycles. Overall, the haematological toxicity of the regimen was acceptable since 14 (16%) and 3 (4%) cycles out of the 86 cycles administered were complicated with grade 3 and 4 neutropaenia, respectively. No grade 4 anaemia or thrombocytopaenia was observed. One patient, at the dose level IV, developed grade 3 anaemia, and another one, at the dose level III, developed grade 3 thrombocytopaenia (Table 4).

The most common non-haematological toxicity was grade 2 asthenia complicating 19% of the cycles; the incidence of other non-haematological toxicities were rare (<5%) and mild (⩽grade 2; Table 3). There was no clinically relevant moderate or severe PPE while there was no patient who presented a reduction of LVEF >10% of the baseline values or congestive heart failure. Owing to the toxicities observed, 21 (24%) of the treatment cycles were delayed and another 6 (7%) were given with a dose reduction. The median duration of treatment delay was 9 days (range, 4–31 days) for all cycles. The median cumulative dose administered was 604 mg m^−2^ (range, 111–1800 mg m^−2^) for PCX, 71 mg m^−2^ (range, 11.9–186 mg m^−2^) for PLD and 5564 mg m^−2^ (range, 1000–14 376 mg m^−2^) for GEM. All patients have discontinued treatment for the following reasons: progressive disease (*n*=9 patients), completion of treatment (*n*=9 patients), consent withdrawn (*n*=2 patients) and neutropaenia (*n*=2 patients).

### Pharmacokinetics

The effects of dose escalation on the PK parameters of the combination are shown in Table 5. The PKs of PLD were characterised by *C*_max_ ranging from 5.51 to 7.78 mg l^−1^, with a typically slow post-infusional elimination with half-life values ranging between 38.52 and 63.44 h, and CL from 0.018 to 0.059 l h^−1^. The areas under the curve for all time points (AUC_all_) were similar to those extrapolated to infinity (AUC_inf_) (range: 208.07–514.18 and 222.02–670.12 mg h l^−1^, respectively), while the value of *V*_z_ ranged between 1.65 and 3.62 l. PK of PCX were defined by *C*_max_ varying from 0.96 to 1.65 mg l^−1^, detected at the end of the drug infusion (*t*_max_, 3 h), and by an elimination with *t*_1*/*2_ and CL values ranging from 2.82 to 5.09 h and 0.016 to 0.028 l h^−1^, respectively; the AUC_all_ estimated from concentration–time data were between 3.67 and 6.18 mg h l^−1^ (Table 5). The PK profile of GEM presented a dose escalated change from level II to III of *C*_max_ (4.46–12.27 mg l^−1^) achieved at the end of infusion (*t*_max_, 0.50 h), AUC_all_ (2.95–7.26 mg h l^−1^), AUC_inf_ (2.66–6.80 mg h l^−1^) and CL (323.64–160.01 l h^−1^).

### Response to treatment

Eighteen patients with bidimensionally measurable disease were evaluable for response. One patient achieved a partial response and nine patients stable disease. The partial response occurred in a patient with carcinoma of unknown primary site receiving first-line treatment at the dose level III. The duration of response was 8.7 months and the median time to tumour progression for the whole group of patients was 4.5 months (range, 1.0–16.5 months).

Regarding the nine breast cancer patients who were enrolled onto the study, they received the study regimen as first-line (*n*=2 patients), second-line (*n*=2 patients) and third-line treatment (*n*=5 patients). Additionally, none of the four non-evaluable for response patients discontinued treatment due to disease progression. Two of them discontinued due to neutropaenia and the other two, although have completed the treatment, were considered non-evaluable for response because they had non-measurable disease.

## DISCUSSION

Doxorubicin is one of the most widely used anticancer drugs but despite its excellent antitumour activity, its use is limited by drug-associated toxicities, particularly myelosuppression and cardiotoxicity. The combination of PCX and anthracyclines has been also evaluated in several trials using a variety of doses and administration schedules, with studies showing drug interaction with respect to disposition and toxicity. Indeed, the PK studies of doxorubicin in regimens containing PCX have demonstrated that the schedule-dependent increase in *C*_max_ and AUC and the reduction in doxorubicin clearance were associated with severe neutropaenia and mucositis (Holmes *et al*, 1996) as well as cardiac toxicity (Gianni *et al*, 1997). Furthermore, the addition of GEM to the PCX/anthracycline combination was associated with an increased incidence of severe neutropaenia ranging from 62 to 72% of patients (Sanchez-Rovira *et al*, 2000; Conte *et al*, 2001; Cappuzzo *et al*, 2004; Zielinski *et al*, 2005).

In order to improve the toxicity profile of the combination of PCX with anthracyclines, PLD was substituted for doxorubicin or epirubicin and the regimen was administered on a biweekly basis; this phase I study indicated that the regimen was very well tolerated with treatment delay due to grade 2 and 3 neutropaenia to be the dose-limiting event (Mavroudis *et al*, 2002). Based on the favourable toxicity profile of the biweekly administration of PCX and PLD and in an effort to further develop this schedule, GEM was added to the PCX/PLD combination in this phase I trial to determine the MTD and the DLT of the combination. Grade 4 neutropaenia (two cases) and grade 2/3 neutropaenia and anaemia resulting in treatment delays on days 15 or 28 were the dose-limiting events observed in this study. Accordingly, the recommended doses for future phase II studies were PLD 12 mg m^−2^ on day 1 and PCX 100 mg m^−2^ followed by GEM 1000 mg m^−2^ on day 2 administered every 2 weeks without growth factor support.

In the present study, there was no need for the prophylactic use of growth factor since clinically relevant neutropaenia was rare. Conversely, in a study by Sanchez-Rovira *et al* (2000) almost 60% of the cycles required support with granulocyte colony-stimulating factor. In addition, none of the patients in our study developed febrile neutropaenia. Grade 2 asthenia was the most common non-haematological toxicity resulting in the discontinuation of treatment in one patient. However, since the majority of patients experienced progressive disease, it is not clear whether asthenia was attributed exclusively to the chemotherapy regimen.

Skin and mucosal toxicities are frequent adverse events of PLD. These adverse events are dose- and schedule-related with stomatitis being more frequent and severe at higher doses, while shorter dosing intervals lead to an increased incidence and severity of skin manifestations. However, in the present study, mucositis, a relatively frequent adverse event of PCX plus anthracycline plus GEM combination (Sanchez-Rovira *et al*, 2000; Conte *et al*, 2001; Zielinski *et al*, 2005), as well as PPE, were mild and uncommon. Moreover, neurotoxicity or cardiac toxicity, which already have been reported to complicate the PCX plus epirubicin plus GEM regimen (Sanchez-Rovira *et al*, 2000; Zielinski *et al*, 2005), were not observed in the present study.

This favourable toxicity profile of the PLD/PCX/GEM combination should be, mainly, attributed to both the substitution of free anthracycline for PLD and the low cumulative dose of PLD. Indeed, its formulation protects the liposomes from detection by the mononuclear phagocyte system, thus increasing its circulation time and allowing for more targeted delivery of doxorubicin to the tumour cells (Working *et al*, 1994). The comparison of PLD to conventional doxorubicin, as first-line treatment of patients with MBC revealed that the two drugs were broadly comparable in terms of efficacy, but PLD had a different safety profile with significantly reduced cardiac toxicity including those subgroups of patients at increased risk of developing a cardiac event; conversely, there was a higher incidence of skin toxicity (PPE) and less alopecia, nausea, vomiting and myelosuppression with PLD than with conventional doxorubicin (O'Brien *et al*, 2004).

The PK parameters of 13 patients were studied in order to evaluate the PK profile of different dose levels of the PLD/PCX/GEM combination. PK analysis demonstrated that the studied combination did not produce any major changes since all studied parameters were found to be in accordance with other published reports (Lyass *et al*, 2000; Ichiki *et al*, 2003). Patients at dose level III presented significant changes in some PK parameters of PLD that cannot be associated with the administration of the other two drugs since that effect appeared on PLD-monotherapy day (first day of the cycle) and before PCX and GEM administration (PLD *t*_max_ for dose level III: 3 h). Studies on gender-dependent differences of doxorubicin have already been reported (Clements *et al*, 2002; Suzuki *et al*, 2006). In the present study, five out of six patients at dose level III were females implying that gender-related effects could be the most possible explanation for the observed differences in the PK parameters of PLD. Finally, dose escalation of GEM (800–1000 mg m^−2^) and PCX (100–110 mg m^−2^) were the reasons for the PK changes observed between dose levels II and III/IV for GEM and dose levels III and IV for PCX (Table 5). Based on the above PK data, no obvious drug–drug interactions were observed.

Based on previous observations of considerable activity and limited toxicity of GEM as first-, second- and even third-line treatment in phase II studies of MBC (Seidman, 2003), the addition of GEM to epirubicin and PCX was speculated to increase activity of the combination without additional toxicity. Thus, a combination regimen of GEM, epirubicin and PCX (GET) was developed, which has shown a response rate of 92% in a phase II study of patients with MBC (Conte *et al*, 2001). However, in a recent phase III trial the GET regimen failed to demonstrate superiority over FEC and at the same time had a higher toxicity (Zielinski *et al*, 2005). Moreover, combination chemotherapy has resulted in similar efficacy as sequential single-agent therapy in MBC (Sledge *et al*, 2003). Therefore, it remains to be tested if any triplet combination is superior to the sequential administration of the corresponding single agents.

The low-response rate observed in our study (only 1 in 18 evaluable patients presented a partial response) may be due to the advanced line of treatment, since most of the patients were heavily pretreated, as well as to the short treatment period (median of three cycles/patient).

In conclusion, the current phase I study clearly demonstrates that the triplet combination of PLD plus PCX and GEM given every 2 weeks is a well-tolerated regimen, which merits further evaluation in phase II studies in patients with sensitive tumours such as breast, ovarian or head and neck cancer. However, any clinical application of this regimen should be weighted against the increased cost of treatment.

## Figures and Tables

**Table 1:** Patients' characteristics

	**No. of patients**	**(%)**
Patients enrolled	22	
Evaluable for toxicity	22	
Evaluable for response	18	
		
*Age (years)*		
Median (range)	64 (40–77)	
		
*Gender*		
Male/female	8/14	36/64
		
*Performance status (WHO)*		
0	7	32
1	13	59
2	2	9
		
*Previous chemotherapy regimens*		
0	6	27
1	6	27
2	10	46
		
*Previous treatment*		
Surgery	18	82
Adjuvant chemotherapy	8	36
Neo-adjuvant chemotherapy	1	5
Chemotherapy for metastatic disease	16	73
Adjuvant radiotherapy	7	32
Radiotherapy for metastatic disease	4	18
None	1	5
		
*Type of tumour*		
Breast cancer	9	41
Lung cancer	2	9
Bladder cancer	3	14
Ovarian cancer	2	9
Cancer of unknown primary	1	5
Other	5	23

**Table 2:** Dose escalation levels, number of patients enrolled and DLTs during the first cycle

**Dose level**	**PLD (mg m^−2^)**	**PCX (mg m^−2^)**	**GEM (mg m^−2^)**	**Patients enrolled**	**DLT (number of patients)**
I	10	100	800	3	—
II	12	100	800	6	Grade 3 neutropaenia^a^ (1)
					Grade 2 neutropaenia^a^ (1)
III	12	100	1000	9	Grade 4 neutropaenia (1)
					Grade 2 anaemia^a^ (1)
IV	12	110	1000	4	Grade 4 neutropaenia (1)
					Grade 3 neutropaenia^a^ (1)
					Grade 2 neutropaenia^a^ (1)

DLT=dose-limiting toxicity.

a The toxicity was considered a ‘DLT’ because it resulted in treatment delay.

**Table 3:** Cycles of chemotherapy complicated by grade 2–4 haematological and non-haematological toxicities

**Dose level**	**Cycles**	**Neutropaenia grade 2/3/4**	**Anaemia grade 2/3/4**	**Thrombocytopaenia grade 2/3/4**	**Nausea/vomiting grade 2/3/4**	**Diarrhoea grade 2/3/4**	**Mucositis grade 2/3/4**	**Neurotoxicity grade 2/3/4**	**Asthenia grade 2/3/4**
I	15	1/2/−	4/−/−	−/−/−	−/−/−	−/−/−	−/−/−	−/−/−	6/−/−
II	22	6/4/−	4/−/−	−/−/−	1/−/−	2/1/−	1/−/−	−/−/−	−/−/−
III	36	7/5/2	5/−/−	−/1/−	3/−/−	−/−/−	2/−/−	3/−/−	10/−/−
IV	13	3/3/1	2/1/−	1/−/−	−/−/−	−/−/−	−/−/−	−/−/−	−/−/−

**Table 4:** Worst (grades 2–4) haematological and non-haematological toxicities per patient during all cycles

**Dose level**	**Patients**	**Neutropaenia grade 2/3/4**	**Anaemia grade 2/3/4**	**Thrombocytopaenia grade 2/3/4**	**Nausea/vomiting grade 2/3/4**	**Diarrhoea grade 2/3/4**	**Mucositis grade 2/3/4**	**Neurotoxicity grade 2/3/4**	**Asthenia grade 2/3/4**
I	3	−/2/−	2/−/−	−/−/−	−/−/−	−/−/−	−/−/−	−/−/−	1/−/−
II	6	1/2/−	3/−/−	−/−/−	1/−/−	2/1/−	1/−/−	−/−/−	−/−/−
III	9	2/2/1	3/−/−	−/1/−	3/−/−	−/−/−	1/−/−	2/−/−	4/−/−
IV	4	1/2/1	−/1/−	1/−/−	−/−/−	−/−/−	−/−/−	−/−/−	−/−/−

**Table 5:** Means (±s.d.) of pharmacokinetic parameters at different dose levels of the PLD/PCX/GEM combination

**Dose levels (no. of patients)**		**I (2)**	**II (2)**	**III (6)**	**IV (3)**
PLD	*t*_max_ (h)	1.0	1.0	3.0	4.3
	*C*_max_ (mg l^−1^)	6.30±1.94	5.51±0.36	7.78±1.42	6.85±0.64
	*t*_1/2_ (h)	38.52±2.11	47.16±11.07	63.44±11.67	48.75±14.03
	AUC_all_ (mg h l^−1^)	208.07±68.76	210.40±72.03	514.18±102.24	428.81±89.28
	AUC_inf_ (mg h l^−1^)	222.02±71.57	241.90±95.05	670.12±100.52	501.36±99.47
	*V*_z_ (l)	2.84±1.05	3.62±0.63	1.65±0.27	1.78±0.44
	CL (l h^−1^)	0.050±0.016	0.059±0.023	0.018±0.003	0.025±0.005
					
PCX	*t*_max_ (h)	3.0	3.0	3.0	3.0
	*C*_max_ (mg l^−1^)	1.65±0.12	0.96±0.05	1.16±0.34	1.46±0.06
	*t*_1/2_ (h)	5.09±0.43	2.82±1.64	3.55±1.62	4.19±1.49
	AUC_all_ (mg h l^−1^)	6.18±0.75	3.67±0.62	4.50±1.03	5.83±0.29
	AUC_inf_ (mg h l^−1^)	6.49±0.88	3.70±0.71	4.58±1.11	5.94±0.62
	*V*_z_ (l)	0.114±0.006	0.101±0.044	0.112±0.051	0.109±0.031
	CL (l h^−1^)	0.016±0.002	0.028±0.005	0.023±0.005	0.019±0.002
					
GEM	*t*_max_ (h)	ND	0.50	0.50	0.58 (0.50–0.75)
	*C*_max_ (mg l^−1^)	ND	4.46±0.80	12.27±3.28	10.46±2.23
	*t*_1/2_ (h)	ND	0.26±0.07	0.36±0.18	0.48±0.35
	AUC_all_ (mg h l^−1^)	ND	2.95±1.02	7.26±1.89	8.55±1.08
	AUC_inf_ (mg h l^−1^)	ND	2.66±0.71	6.80±1.65	8.15±1.72
	*V*_z_ (l)	ND	111.84±0.74	91.75±78.04	77.80±41.38
	CL (l h^−1^)	ND	323.64±86.33	160.01±54.59	128.03±25.80

GEM=gemcitabine; ND=not determined; PCX=paclitaxel; PLD=pegylated liposomal doxorubicin.
